# Coding-complete genome sequence of a divergent black queen cell virus strain identified from honeybees (*Apis mellifera*) in Romania

**DOI:** 10.1128/mra.00122-26

**Published:** 2026-05-06

**Authors:** Mariyam Mustajab, Nikolas Basler, Lina De Smet, Dirk C. de Graaf, Jelle Matthijnssens

**Affiliations:** 1 Laboratory of Clinical and Epidemiological Virology, Department of Microbiology, Immunology and Transplantation, Rega Institute for Medical Research – KU Leuven 54515 , Leuven, Belgium; 2 Department of Biochemistry and Microbiology, Laboratory of Molecular Entomology and Bee Pathology, Ghent University 366761, Ghent, Belgium; DOE Joint Genome Institute, Berkeley, California, USA

**Keywords:** black queen cell virus, *Apis mellifera*, viral metagenomics

## Abstract

We report the 8,451-nt coding-complete genome sequence of a divergent black queen cell virus strain (BQCV-Ro). This strain was identified via retrospective analyses of metagenomic virome data of *Apis mellifera* samples from Europe. The genome assembly is supported by extensive read mapping, with 100% horizontal coverage and a mean sequencing depth of ~38,171×. and shares 94.3% identity with the BQCV Yeongdeok isolate.

## ANNOUNCEMENT

Black queen cell virus (BQCV), a member of the genus *Triatovirus* (family *Dicistroviridae*), is a major viral pathogen of the honeybee (*Apis mellifera*) (1). While often asymptomatic in adult bees, it causes mortality in queen larvae at elevated titers and is characterized by the darkening of the queen cell walls (2). Here, we describe a divergent BQCV strain (BQCV-Ro) identified through the re-analysis of a metagenomic data set of honeybees originally collected in the framework of the EU-funded B-GOOD project (3).

The sample was derived from an apparently healthy Romanian honeybee hive located at latitude 46.7598 and longitude 23.574, collected on 17 July 2020. The guts of 10 worker bees were dissected, and their rectums were pooled before the sample was processed following the NetoVIR protocol for the enrichment of both DNA and RNA viral particles (4). In short, the sample was homogenized (MINILYS, Bertin Technologies) at 3,000 rpm for 1 min with 2.8 mm zirconium oxide beads (Precellys), centrifuged at 17,000 × *g* for 3 min, passed through a 0.8 µm PES filter, and treated with nuclease to remove host and environmental nucleic acids. RNA and DNA were extracted using the QIAamp Viral RNA Mini Kit (QIAGEN) without carrier RNA. The extract underwent reverse transcription and random amplification (WTA2, Merck), and the resulting cDNA was processed into Illumina sequencing libraries using the Nextera XT kit (Illumina) before sequencing on the NovaSeq 6000 (Illumina) platform using 150 bp paired-end reads. Raw paired-end reads were quality-filtered and adapter-excised via Trimmomatic v.0.39 (5). Host-derived sequences were computationally subtracted by aligning reads to the *Apis mellifera* reference genome (GCF_003254395.2) using Bowtie2 v.2.5.1 (6). *De novo* assembly of the remaining reads was performed using metaSPAdes v3.15.3. Taxonomic classification of the obtained contigs was achieved using DIAMOND v2.0.9 against the NCBI non-redundant (nr) database (downloaded on 17 March 2023), supplemented by BLASTn searches against the NCBI nucleotide (nt) database (downloaded on 17 March 2023). Unless otherwise specified, default parameters were used for all software tools. Strain BQCV-Ro was identified in a honeybee colony in Romania during the summer and possesses a coding-complete genome of 8,451 nucleotides with a GC content of 40.2%, consistent with the 34–45% range characteristic of *Dicistroviridae* members (7). Read mapping revealed 100% horizontal coverage with a mean depth of approximately 38,171×. Open reading frames (ORFs) were predicted *de novo* using NCBI ORFfinder (standard genetic code; minimum length 300 nt), identifying two major ORFs separated by a 316 nt intergenic region. BLASTp analysis against the NCBI nr database showed that ORF1 (4,887 nt) shared 98.4% amino acid identity (100% coverage) with a black queen cell virus non-structural polyprotein (accession WIL00273.1), while ORF2 (2,454 nt) showed 98.7% identity (100% coverage) to a structural polyprotein (accession ABS82440.1). A BLASTn analysis of the complete sequence demonstrated 94.3% nucleotide identity (100% query coverage) to the BQCV Yeongdeok strain from the Republic of Korea (GenBank accession OR496416.1). The characterization of this sequence expands the documented genetic landscape of *Apis* pathogens in Eastern Europe and underscores the efficacy of mining extant metagenomic data sets for the identification of circulating viral strains.

## Data Availability

The black queen cell virus genome sequence has been deposited in GenBank under accession number PX926923. The raw sequencing reads have been deposited in the NCBI Sequence Read Archive (SRA) under accession number SRR34920129, as part of BioProject PRJNA1302169 (BioSample SAMN50464891).

## AFTER EPUB

*[This article was published on 6 May 2026 with an error in the Abstract. The Abstract was corrected in the current version, posted on 11 May 2026.]*
